# Delirium: Clinical Presentation and Outcomes in Older COVID-19 Patients

**DOI:** 10.3389/fpsyt.2020.586686

**Published:** 2020-11-12

**Authors:** Renzo Rozzini, Angelo Bianchetti, Francesca Mazzeo, Giulia Cesaroni, Luca Bianchetti, Marco Trabucchi

**Affiliations:** ^1^Geriatric Department, Fondazione Poliambulanza Istituto Ospedaliero, Brescia, Italy; ^2^Associazione Italiana di Psicogeriatria, Brescia, Italy; ^3^Medicine and Rehabilitation Department, Istituto Clinico S. Anna Hospital, Brescia, Italy; ^4^Geriatric Rehabilitation Unit, Anni Azzurri, Brescia, Italy

**Keywords:** COVID 19, delirium, elderly, frailty, mortality

## Abstract

The aim of the study is to describe the clinical characteristics and outcomes of a series of older patients consecutively admitted into a non-ICU ward due to SARS-CoV-2 infection (14, males 11), developing delirium. Hypokinetic delirium with lethargy and confusion was observed in 43% of cases (6/14 patients). A total of eight patients exhibited hyperkinetic delirium and 50% of these patients (4/8) died. The overall mortality rate was 71% (10/14 patients). Among the four survivors we observed two different clinical patterns: two patients exhibited dementia and no ARDS (acute respiratory distress syndrome), while the remaining two patients exhibited ARDS and no dementia. The observed different clinical patterns of delirium (hypokinetic delirium; hyperkinetic delirium with or without dementia; hyperkinetic delirium with or without ARDS) identified patients with different prognosis: we believe these observations may have an impact on the management of older subjects with delirium due to COVID-19.

## Introduction

Although the most frequent and life-threatening complications of coronavirus disease 19 (COVID-19) are respiratory, there are increasing reports of neurological and psychiatric involvement (1).

It is known that delirium can be the symptom of the presentation of many diseases, particularly in frail and older patients, and is recognized as an independent risk factor for mortality (2). The overall prevalence of delirium in the hospital setting is about 14–24%; its prevalence is higher, about 30%, in emergency, surgical, or medical wards (3, 4). To date, the clinical presentation of delirium in older patients with COVID-19 infection have rarely been described; in fact, although some studies focus on epidemiological data and outcome, few studies analyze the clinical aspects of delirium in COVID-19 (5–8). The aim of this study is to describe clinical characteristics and outcomes of a series of elderly patients presenting delirium as the main symptom of COVID-19.

## Materials and Methods

The study was carried out in the COVID ward in an Acute Care Hospital located in Brescia, one of the hardest hit cities by SARS-CoV-2 infection in northern Italy (9). We collected the characteristics of 14 older patients (age range 70–90, mean age 78.2; 11 males) consecutively admitted developing prevalent or incident delirium (respectively 10 and 4 cases). All the patients were admitted with a diagnosis of COVID-19, confirmed by a real-time reverse-transcriptase-polymerase chain reaction (rRT-PCR); three patients came from nursing homes, the remainder from home.

Medical information collected were age, sex, PaO_2_/FiO_2_, chest x-ray or CT, comorbidities [ischemic heart disease, chronic obstructive pulmonary disease (COPD), hypertension, diabetes, malignancies, neurodegenerative diseases], blood tests [hemoglobin, platelets count, neutrophils, lymphocytes, C-reactive protein (CRP), urea, and creatinine], and oxygen therapy (i.e., from nasal cannula to high flow cannula oxygen therapy to non-invasive ventilation). To assess the severity of COVID-19 pneumonia the SIAARTI criteria were followed, i.e., *mild ARDS* (acute respiratory distress syndrome): PaO_2_/FiO ratio 201–300; *moderate ARDS*: 101–200, and s*evere ARDS*: ≤ 100 (10). The diagnosis of dementia was made on the basis of the data collected from clinical records, while the severity of dementia was assessed by CDR (11) and functional status by the Barthel Index (12). CDR was estimated based on information collected from family members and the records of patients. Delirium was detected through 4At (assessment test for delirium and cognitive impairment) (13).

Clinical criteria were used to characterize delirium subtypes: hypoactive or hyperactive. The presence of a disturbance of consciousness was retrospectively defined by altered arousal.

## Results

Hyperactive delirium with aggression and agitation was observed in eight patients, while the remaining six patients exhibited hypoactive delirium with lethargy and confusion.

Moreover, dementia was diagnosed in six out of 14 patients; among these, four developed hypokinetic delirium, while the remaining two developed hyperkinetic delirium. Patients without dementia were younger, with a mean age of 74.1 years (see Table 1).

**Table 1:** Characteristics and outcomes of 14 older patients with confirmed diagnosis of COVID-19 and delirium.

**Age range**	**From**	**CDR/Type of dementia**	**BoA**	**Comorbidities**	**Symptoms and signs**	**CT scan**	**PaO_**2**_/FiO_**2**_**	**Delirium**	**Drugs**	**Outcomes**
90–94	NH	2 AD+VD	10	AF Lung cancer Previous venous thrombosis	Dyspnea RR 24	NA	245	Drowsiness (on admission)	–	Died (day 10)
80–84	NH	3 AD+VD	10	CHD Hypertension T2DM CKD Previous stroke Epilepsy	Fever Cough Dyspnea RR 36	NA	269	Drowsiness (on admission)	Trazodone (previous)	Died (day 12)
80–84	NH	4 AD	0	Hypertension UTI	Fever Dyspnea RR 36	NA	255	Drowsiness (on admission)	–	Died (day 5)
90–94	Home	3 AD+VD	0	HF CHD AF Previous stroke Gastritis	Fever Dyspnea Confused state RR 20	75%	261	Drowsiness (on admission)	Brotizolam (previous)	Died (day 2)
75–79	Home	0	100	T2DM Obesity	Fever Dyspnea Syncope RR 26	50–75%	148	Drowsiness (on admission)	None	Died (day 3)
75–79	Home	0	100	AF Hypertension T2DM	Cough Dyspnea RR 28	75%	136	Drowsiness (on admission)	None	Died (day 1)
70–74	Home	0	100	CHD Hypertension T2DM	Fever Dyspnea RR 20	75%	220	Agitation (day 2)	Quetiapine Lorazepam Haloperidol	Died (day 3)
70–74	Home	0	100	HF AF Hypertension T2DM	Fever Cough Dyspnea RR 17	75%	254	Agitation (day 2)	None	Died (day 5)
75–79	Home	0	100	Hypertension T2DM Peripheral artery disease	Cough Dyspnea RR 28	75%	260	Agitation (on admission)	Diazepam	Died (day 2)
70–74	Home	0	55	AF Hypertension T2DM Previous stroke Depression	Fever RR 16	75%	252	Agitation (on admission)	–	Died (day 10)
75–79	Home	2 AD+VD	40	AF Hypertension	Dyspnea RR 28	<25%	330	Agitation (day 2)	Haloperidol Quetiapine	Discharged (day 23)
85–89	Home	2 AD+VD	20	CHD Hypertension Gastritis	Confused state Falls RR 16	<25%	320	Agitation (on admission)	Citalopram, Haloperidol	Discharged (day 3)
70–74	Home	0	45	Hypertension T2DM	Fever Cough Dyspnea RR 22	75%	126	Agitation (on admission)	Quetiapine	Discharged (day 4)
75–79	Home	0	60	Hypertension PD Previous stroke	Dyspnea RR 20	50–75%	179	Agitation (day 2)	Lorazepam	Discharged (day 13)

*NA, not available; AD, Alzheimer's disease; VD, vascular dementia; CDR, Clinical Dementia Rating Scale; BoA, Barthel Index score on admission; HF, heart failure; CHD, coronary heart disease; AF, atrial fibrillation; T2DM, type 2 diabetes mellitus; CKD, chronic kidney disease; PD, Parkinson disease; RR, respiratory rate; CT scan, visual quantitative evaluation of the acute lung inflammatory lesions involving each lobe*.

The drugs used to treat patients with hyperkinetic delirium were: lorazepam (2 cases), diazepam (1 cases), quetiapine (3 cases), and haloperidol (3 cases). Two patients with hypokinetic prevalent delirium were treated before hospitalization with brotizolam (1 case) and trazodone (1 case).

Two of the patients were hospitalized for stage III pneumonia (PaO_2_/FiO_2_ ratio >300), eight patients were hospitalized for stage IV pneumonia-mild ARDS (200<PaO_2_/FiO_2_ <3008), and four patients were hospitalized with stage IV pneumonia-moderate ARDS (100<PaO_2_/FiO_2_ <200).

Upon admission, the patients presented the following symptoms: fever (7 cases), dyspnea (12 cases), cough (4 cases), fall and syncope (one case).

Almost all the patients (12/14) had a respiratory rate greater than 19.

The overall mortality rate was 71% (10/14 patients). All 6 of the patients exhibiting hypokinetic delirium and the 50% of patients (4/8) with hyperkinetic delirium died. Patients with hypokinetic delirium exhibited dementia and mild ARDS in four cases and no dementia and moderate ARDS in two cases.

Among the four survivors we observed two different clinical patterns: two patients exhibited dementia and no ARDS, while the remaining two patients exhibited ARDS and no dementia.

All patients living in a nursing home developed hypokinetic delirium and died.

A chest CT scan was taken for 11 of the patients: in two cases the lung involvement was less than 25%, in two cases it was from 50 to 75%, and in seven cases it was greater than 75%. The two cases with lower lung involvement survived; one patient with intermediate (50–75%) and one with greater involvement (>75%) also survived.

Each patient showed a high number of comorbidities: nine patients were affected by cardiovascular diseases (mainly coronary heart disease, atrial fibrillation, and heart failure), 12 by hypertension, and eight by diabetes. In particular, only four patients had no more than two comorbid conditions. In detail: survivors with hyperkinetic delirium had two or three comorbidities; deceased patients with hyperkinetic delirium had three or more comorbidities; deceased patients with hypokinetic delirium had two comorbidities in two cases and three or more in four cases.

## Discussion

With increasing frequency, delirium is reported as a symptom of the presentation of COVID-19 in older patients, although clinical aspects are rarely characterized (14). In a French series of elderly patients with COVID-19, delirium was present in 26.7% of patients, in two thirds of the cases in the hypokinetic form (15). In a series of hospitalized older patients with COVID-19 in the UK, delirium was observed in 25.2% of the sample (16). In older patients with dementia, delirium was a clinical manifestation of COVID-19 in 67% of cases, in 75% of these cases in the hypokinetic form (17). Mortality rates in these case series related to COVID-19 disease are still inconclusive and so comparison with other literature is uncertain.

In our patients with delirium, mortality was higher (71%) than previously reported for cases of hospitalized older people with delirium (ranging from 9 to 25%) (4). All subjects who developed hypokinetic delirium died. According to the literature, this form of delirium is associated with worse outcomes, particularly among patients affected by dementia (18). Multimorbidity is a condition associated with higher mortality, especially among patients who developed hypokinetic delirium: thus, hypokinetic delirium needs to be considered a marker of poor prognosis even in previously fit patients (3).

The onset of delirium is due to a complex interaction between the baseline vulnerability of the patient or predisposing factors and noxious insults or precipitating factors; recent observations lead us to believe that frailty and immunosenescence constitute factors that explain the excess mortality in elderly subjects with COVID-19 (19).

In our study, hyperkinetic delirium in cognitively unimpaired patients with mild ARDS had a better prognostic value than hypokinetic delirium in those with the same lung impairment. Hyperkinetic delirium in patients with dementia was observed in non-ARDS pneumonia (PaO_2_/FiO_2_ > 300). Patients with hyperkinetic delirium who died had a higher noxious insult (i.e., 200 < PaO_2_/FiO_2_ < 300) or dementia, and high level of comorbidities.

The high mortality rate of subjects developing delirium as an onset symptom of COVID-19, particularly in its hypokinetic form, could suggest brain involvement rather than the worsening effect of a pre-existing condition of frailty. Taking cognizance of the emergency due to the outbreak of COVID-19 and the consequent necessity of brief and easy-to-use tools and the involvement of non-expert doctors and nurses in COVID wards, to diagnose delirium we decided to use the 4AT test, a reliable tool designed for delirium detection in clinical practice (13).

Based on our observations, we hypothesize that delirium subtypes may be markers of biological severity of precipitating disease in COVID-19 patients. Specifically, patients suffering from a higher involvement of brain function and thus manifesting hypokinetic delirium, have a worse prognosis, while those who develop hyperkinetic delirium with a lower degree of dysregulation induced by the disease have a better chance of survival. Data on the ARDS stage confirm this interpretation since deceased patients with hypokinetic delirium and dementia were the most biologically compromised (with the most severe form of ARDS).

These different clinical patterns (hypokinetic delirium; hyperkinetic delirium with or without dementia; hyperkinetic delirium with or without ARDS) identify patients with different prognosis. Although the data were collected in a relatively limited number of cases, these observations may have an impact on the management of older subjects with delirium due to COVID-19.

In conclusion, our study indicates that delirium, particularly in the hypokinetic form, is related to a high risk of mortality in patients with COVID-19, especially in the presence of dementia. Therefore, a systematic recognition of this syndrome in COVID-19 patients is crucial for establishing a reliable prognosis.

## Data Availability Statement

All datasets presented in this study are included in the article/Supplementary Material.

## Ethics Statement

The studies involving human participants were reviewed and approved by Comitato Etico di Brescia. Written informed consent for participation was not required for this study in accordance with the national legislation and the institutional requirements.

## Author Contributions

RR, FM, and GC contribute to evaluation of cases, data management and discussion. AB, LB, and MT reviewed and discussed the manuscript. AB and RR wrote the first draft. All authors carefully reviewed, discussed and contributed to various draft of the manuscript. All authors approved the final manuscript.

## Conflict of Interest

The authors declare that the research was conducted in the absence of any commercial or financial relationships that could be construed as a potential conflict of interest.
